# Coronavirus (SARS-CoV-2) in gastroenterology and its current epidemiological situation: An updated review until January 2021

**DOI:** 10.17179/excli2021-3417

**Published:** 2021-02-16

**Authors:** Ahmed Nabil, Mohamed M. Elshemy, Koichiro Uto, Reham Soliman, Ayman A. Hassan, Gamal Shiha, Mitsuhiro Ebara

**Affiliations:** 1 Research Center for Functional Materials, National Institute for Materials Science (NIMS), 1-1Namiki, Tsukuba, Ibaraki 305-0044, Japan; 2 Biotechnology and Life Sciences Department, Faculty of Postgraduate Studies for Advanced Sciences (PSAS), Beni-Suef University, Beni-Suef, Egypt; 3 Egyptian Liver Research Institute and Hospital (ELRIAH), Sherbin, El Mansoura, Egypt; 4 Faculty of Science, Menoufia University, Menoufia, Egypt; 5 Tropical Medicine Department, Faculty of Medicine, Port Said University, Egypt; 6 Hepatology and Gastroenterology Unit, Internal Medicine Department, Faculty of Medicine, Mansoura University, Egypt; 7 Graduate School of Pure and Applied Sciences, University of Tsukuba, 1-1-1 Tennodai, Tsukuba, Ibaraki 305-8577, Japan; 8 Graduate School of Industrial Science and Technology, Tokyo University of Science, 6-3-1 Niijuku, Katsushika-ku, Tokyo 125-8585, Japan

**Keywords:** COVID-19, SARS-CoV-2, digestive system, anal swab, GI, liver, epidemiology

## Abstract

Coronaviruses are positive-sense single-strand RNA viruses that infect amphibians, birds, and mammals. Coronavirus Disease 2019 (COVID-19) has become a major health problem caused by one of the coronaviruses called severe acute respiratory syndrome coronavirus 2 (SARS-CoV-2). It has spread fast throughout the globe since its first identification in Wuhan, China, in December 2019. Although COVID-19 is principally defined by its respiratory symptoms, it is now clear that the virus can also affect the digestive system causing gastrointestinal (GI) symptoms like diarrhea, loss of appetite, nausea/vomiting, and abdominal pain as a major complaint. GI symptoms could be the initial signs of preceding respiratory signs, carrying a potential for slowed investigation and raised disease transmission opportunities. Various studies recognized the COVID-19 RNA in stool specimens of infected patients, and its viral receptor angiotensin-converting enzyme-2 (ACE-2) is highly expressed in GI epithelial cells. Many cases were reported negative using nasopharyngeal/oropharyngeal swabs and finally, SARS‐CoV‐2 RNA was detected in their anal/rectal swabs and stool specimens. These suggest that COVID-19 can actively infect and replicate in the GI tract. In this review, we elaborate on the close relationship between SARS-CoV-2 and the digestive system, focusing on the current status in the field of COVID-19 in gastroenterology, liver injury, endoscopy, inflammatory bowel disease, imaging, and the potential underlying mechanisms with illustrating the current epidemiological status regarding this pandemic.

## Introduction

Coronaviruses are zoonotic, positive-sense single-strand RNA viruses. In December 2019, an outbreak of COVID-19 was caused by SARS-CoV-2 in Wuhan City, China. The World Health Organization (WHO) announced the coronavirus outbreak as a global pandemic in March 2020 (WHO, 2020[37]).

It was indicated that SARS-CoV-2 may be transmitted between individuals by several different routes; the primary transmission mode is mainly contacted through respiratory droplets generated by breathing, coughing, sneezing, as well as direct contact with the infected subjects or indirect contact, by hand-mediated viral transfer from the contaminated fomites to the nose, mouth, or eyes (La Rosa et al., 2020[21]).

Various researches have confirmed that the gastrointestinal (GI) tract is also a potential route. Once the coronavirus is attached to the spike protein (S), the viral genome penetrates the cells, uses human cell machinery, and creates multiple viral particles to be released to infect other cells (Aguila et al., 2020[1]).

COVID-19 initially appeared to be primarily respiratory, presenting as fever and cough with a rapid decline requiring ventilatory support. Later, GI symptoms (nausea, vomiting, diarrhea, abdominal pain), neurologic symptoms (loss of taste and smell, stroke), and other nonspecific symptomatology have also been noted (Lin et al., 2020[23]). However, with an expanded emphasis on reporting, this went up in some reports with even pure GI symptoms without respiratory manifestations.

In this article, we reviewed the current status of COVID-19 in gastroenterology and hepatology, imaging, and its current epidemiological level.

## Common Symptoms and Gastrointestinal Manifestations in COVID-19

Wang et al. (2020[35]) reported that there are 6 common signs and symptoms that 30 % of the patients have felt, including fever (98.5 %), fatigue (69.9 %), dry cough (59.4 %), anorexia (39.8 %), myalgia (34.8 %), dyspnea (31.1 %). Although respiratory tract manifestations are the most commonly reported symptoms in COVID-19, emerging data suggest that the gastrointestinal tract and liver might also be affected by SARS-CoV-2, on the basis that gastrointestinal epithelial cells and liver cells express angiotensin-converting enzyme 2 (ACE2), the major receptor of SARS-CoV-2 (Qi et al., 2020[31]).

**Figure 1:**
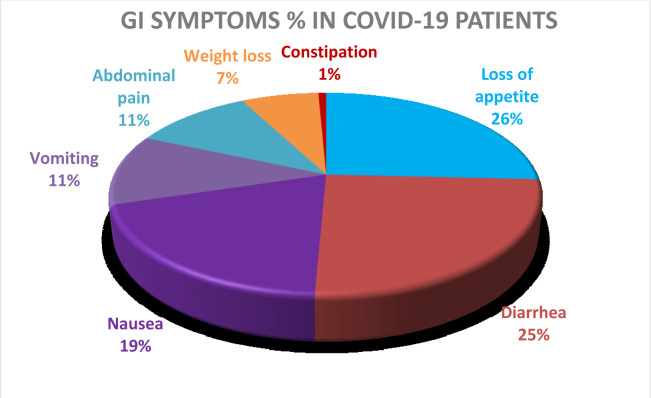
Incidence percentage of different GI symptoms in patients with COVID-19

The most common GI presentation in patients with COVID-19 is diarrhea (3.8 %-34 %), followed by nausea and/or vomiting (3.9 %-10.1 %) and abdominal pain (1.1 %-2.2 %) (Grasselli et al., 2020[15]). Other common GI symptoms reported in patients with COVID-19 are anorexia, anosmia, and dysgeusia (Giacomelli et al., 2020[13]). Figure 1(Fig. 1) shows the incidence percentage of different GI symptoms in patients with COVID-19.

From a total of 35 studies, including 6686 patients with COVID-19, only 29 studies (n=6064) met the inclusion criteria and found GI manifestations in COVID-19 patients at investigation, and the combined prevalence of digestive marks was 15 % (95 % CI 10-21), the most common of which were nausea or vomiting, diarrhea, and anorexia (Mao et al., 2020[25]). Nevertheless, Pan et al. (2020[29]) found that patients with digestive symptoms were more likely to exhibit elevated liver tests, such as AST and ALT, compared with patients without digestive symptoms.

There is accumulating data that bidirectional communication is found between gut and lung, which is termed the gut-lung axis. It is thought that gastrointestinal inflammation causes lung inflammation by this communication. The specific pathway underlying this inflammatory transfer from the gut to the lung is not yet completely revealed (Hufnagl et al., 2020[19]). Intestinal flora is supposed to significantly regulate the development and function of the innate and adaptive immune system, tune the immune cells for pro- and anti-inflammatory responses, and maintain immune homeostasis, thereby affecting the host's susceptibility to various diseases (He et al., 2020[17]). SARS-CoV-2 directly or indirectly harms the digestive system by an inflammatory response. Alterations in the composition and role of the digestive tract flora influence the respiratory tract by the common mucosal immune system and respiratory tract flora diseases similarly affect the digestive tract by immune control. The effect is called the “gut-lung axis” (Budden et al., 2017[4]), which may further explain why patients with COVID-19 pneumonia often have digestive symptoms.

## Proposed Pathophysiologic Mechanism for GI Manifestations of COVID-19

Studies have shown that SARS-CoV-2 can be transmitted through feces (Holshue et al., 2020[18]). Upon infection with COVID-19, it binds to the host cell's angiotensin-converting enzyme 2 (ACE2) receptor, which commonly is found in cilia of glandular epithelium in the gastrointestinal tract and cholangiocytes (Hamming et al., 2004[16]), allows its entry into the target cell and facilitates replication (Xiao et al., 2020[39]). Moreover, it is reported that ACE2 expression is approximately 100-fold higher in the gastrointestinal tract (particularly the colon) than in the respiratory system (Zhang et al., 2020[43]). Therefore, it is not surprising that the digestive system, with several ACE2-expressing organs, would present a risk of being invaded by SARS-CoV-2.

**Figure 2:**
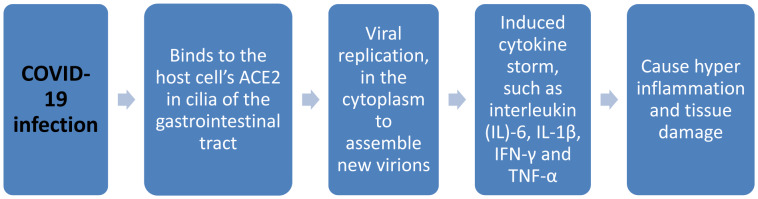
Proposed pathophysiologic mechanisms for GI manifestations of COVID‐19

Viral host receptor ACE2 stained positive mainly in the cytoplasm of gastrointestinal epithelial cells and the cilia of glandular epithelial cells but rarely is expressed in the esophageal squamous epithelial cells. Although viral RNA was also detected in the esophageal mucous tissue, the absence of viral nucleocapsid protein staining in the esophageal mucosa indicates low viral infection in the esophageal mucosa (van Doremalen et al., 2020[34]). After viral entry, virus-specific RNA and proteins are synthesized in the cytoplasm to assemble new virions, which can be released to the gastrointestinal tract (Aguila et al., 2020[1]) (Figure 2(Fig. 2)). Hence, clinicians should give care to GI manifestations and other atypical manifestations of COVID-19 patients to check and cure their infections.

## Current Epidemiological Situation

**Figure 3:**
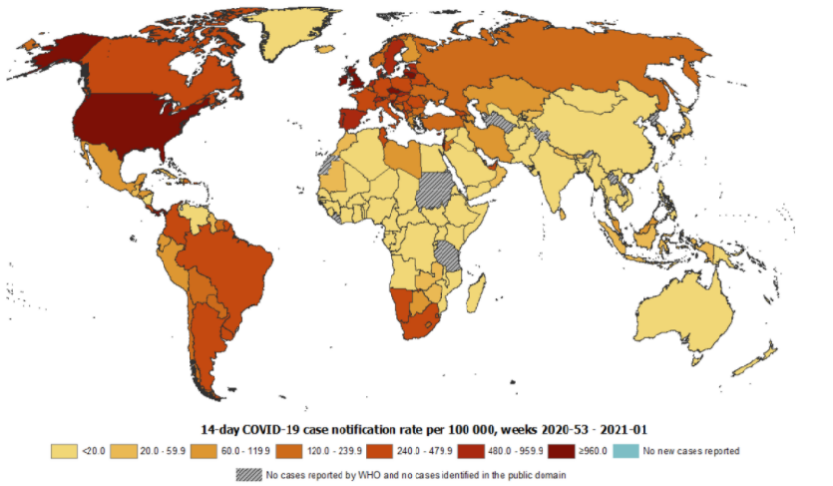
Geographic distribution of the 14-day cumulative number of reported COVID-19 cases per 100 000 population, worldwide, as of 13 January 2021 (ECDC, 2021)

According to the European Centre for Disease Prevention and Control (ECDC), since December 31, 2019 and throughJanuary 13, 2021, there have been 89,802,096 cases of COVID-19 including 1,940,529 deaths. Most cases in America (n = 39,844,634) were reported from: the United States (22,423,006), Brazil (8,131,612), Colombia (1,801,903), Argentina (1,730,908) and Mexico (1,541,633), followed by Europe (n = 28,291,217); most cases reported in Russia (3,425,269), United Kingdom (3,072,349), France (2,783,256), Italy (2,276,491) and Spain (2,111,782), Asia (n = 18,549,010): most cases were in India (10,466,595), Iran (1,286,406), Indonesia (828,026), Iraq (598,369) and Bangladesh (522,453), Africa (n = 3,059,974): most cases were in South Africa (1,231,597), Morocco (452,532), Tunisia (162,350), Egypt (149,792) and Ethiopia (128,616), Oceania (n = 56,556): the largest cases found in Australia (28,614), French Polynesia (17,241), Guam (7,423), New Zealand (2,222) and Papua New Guinea (811) (Figure 3(Fig. 3)). Most deaths in America (n = 925,925) were reported from the United States (374,442), Brazil (203,580), Mexico (134,368), Colombia (46,451) and Argentina (44,654), followed by Europe (n = 623,024) and most deaths were in the United Kingdom (81,431), Italy (78,755), France (67,750), Russia (62,273) and Spain (52,275), Asia (n = 317,547): most deaths were in India (151,160), Iran (56,171), Indonesia (24,129), Iraq (12,844) and Pakistan (10,676), Africa (n = 72,834): most deaths were in South Africa (33,163), Egypt (8,197), Morocco (7,743), Tunisia (5,284) and Algeria (2,807), Oceania (n = 1,193): most deaths were reported in Australia (909), Guam (124), French Polynesia (122), New Zealand (25) and Papua New Guinea (9) (ECDC, 2021[9]).

**Figure 4:**
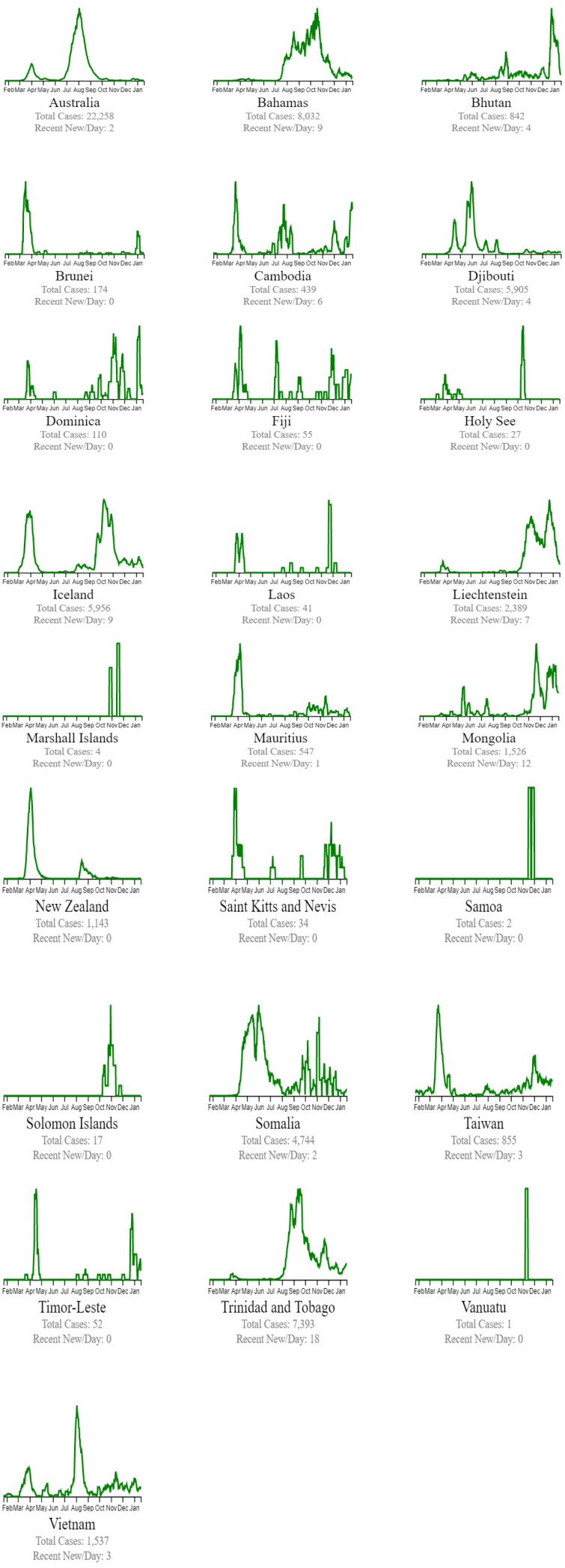
Countries beating COVID-19 (EndCoronavirus, 2021)

**Figure 5:**
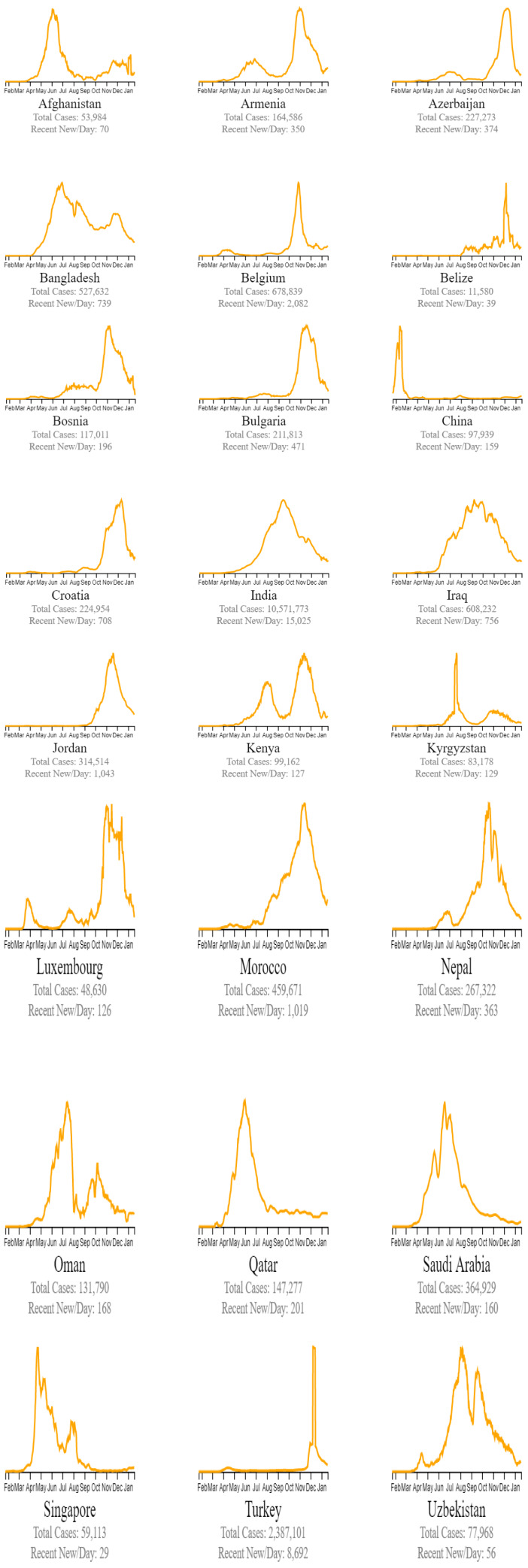
Countries that are nearly there (EndCoronavirus, 2021)

**Figure 6:**
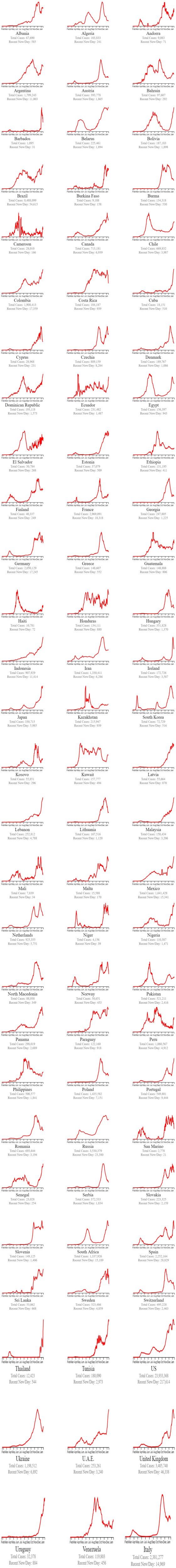
Countries that need to do an action (EndCoronavirus, 2021)

Countries are classified in beating COVID-19 by these three groups: countries beating COVID-19, green plots (Figure 4(Fig. 4)), countries that are nearly there, yellow plots (Figure 5(Fig. 5)), and countries that need to take action red plots (Figure 6(Fig. 6)). These plots are customized for every country to better present the data (EndCoronavirus, 2021[11]).

## COVID‐19 and its Effects on the Digestive System

A recent meta-analysis composed of 60 studies with 4243 patients from China, Singapore, South Korea, the United Kingdom, and the United States showed a pooled prevalence of GI symptoms of 17.6 % including anorexia, nausea, vomiting, diarrhea, and abdominal pain (Cheung et al., 2020[6]). Furthermore, digestive symptoms appeared to be associated with worse outcomes. Whereas about sixty percent of patients without digestive symptoms recovered and were discharged, while only 34.3 % of the patients with digestive manifestations recovered (Pan et al., 2020[29]). Therefore, patients with GI manifestations should attract the attention of both patients and physicians. Table 1(Tab. 1) (References in Table 1: Balaphas et al., 2020[2]; Boettler et al., 2020[3]; Colmenero et al., 2021[7]; Ding et al., 2004[8]; Elli et al., 2020[10]; Garland et al., 2020[12]; Jothimani et al., 2020[20]; Lee-Archer et al., 2020[22]; Macaluso and Orlando, 2020[24]; Monteleone and Ardizzone, 2020[26]; Nabil et al., 2020[27]; Onder et al., 2020[28]; Portincasa et al., 2020[30]; Rana, 2020[32]; RECOVERY Collaborative Group et al., 2020[33]; Wang et al., 2020[36]; Wu et al., 2020[38]; Yang et al., 2010[40]; Yang et al., 2019[41]; Yang et al., 2020[42]) shows the current updated status of COVID-19 in gastroenterology, liver injury, endoscopy, inflammatory bowel diseases (IBD), and imaging.

## Imaging

CT scan works as the screening and diagnostic base for COVID-19: chest imaging in the initial stage presents various plaque shadows and interstitial changes, frequently observed in the peripheral lung and subpleural, and later expanded into multiple ground glass shadows and infiltration shadows in both lungs. In severe cases, lung consolidation can happen, shown as “white lung”, with unique pleural effusion and mediastinal lymph node increase (Chen et al., 2020[5]).

There is a study reporting that 57 % of abdominopelvic CT exams performed on symptomatic COVID-positive patients had positive CT findings in the abdomen or pelvis. Abnormalities in the gastrointestinal tract were the most common (31 %), of which mural thickening was the most frequent. CT findings relating to the gallbladder and biliary system were found in 25 % of patients, including gallbladder distension, mural edema, and findings reported as possible or definite acute cholecystitis; 10 % of patients had biliary ductal dilation. Right upper quadrant ultrasound results previously reported on 37 COVID positive patients showed a similar distribution of pathology, with approximately 60 % demonstrating gallbladder sludge, 3 % demonstrating wall thickening, and 3 % showing pericholecystic fluid (Goldberg-Stein et al., 2020[14]).

## Conclusion

Finally, the COVID-19 pandemic is a highly infectious disease caused by the novel coronavirus SARS-CoV-2 that represents a global public health crisis. Although COVID-19 is principally defined by its respiratory symptoms, it is now clear that the virus can also affect the digestive system causing GI symptoms. ACE2-positive cells in digestive tract tissue strengthen the potential ways for SARS-CoV-2 infection. GI symptoms could be the initial manifestations preceding the respiratory one. In limited scenarios, digestive signs could be the only illness with an absence of any respiratory symptoms, harboring a danger of misdiagnosis. The correlation between the digestive system and COVID-19 deserves further investigation in future relevant studies.

## Notes

Ahmed Nabil, Gamal Shiha (Egyptian Liver Research Institute and Hospital (ELRIAH), Sherbin, El Mansoura, Egypt; Tel: +20 1223280501, E-mail: g_shiha@hotmail.com) and Mitsuhiro Ebara (Research Center for Functional Materials, National Institute for Materials Science (NIMS), 1-1Namiki, Tsukuba, Ibaraki 305-0044, Japan; Tel: 008180-6661-5342, E-mail: EBARA.Mitsuhiro@nims.go.jp) equally contributed as corresponding authors.

## Acknowledgement

All authors express their great gratitude to researchers, physicians, nurses, health care technicians, and all coworkers in the frontlines in Egypt, Japan, and any spot of the globe who spend their lives fighting this virus, hoping this work could help them in their mission.

Special thanks to Ebara Labo., NIMS, Japan research team & ELRIAH, El Mansoura, Egypt researchers, physicians, nurses, and health care technicians.

## Authors’ contributions

Ahmed Nabil: Resources, conceptualization, original draft writing, supervision, review & editing. Koichiro Uto: Original draft writing, review & editing. Mohamed M. Elshemy: Original draft writing, review, editing & resources. Reham Soliman: Writing, review & editing. Ayman A. Hassan: Writing & editing. Gamal Shiha: Conceptualization, original draft writing, review, editing & supervision. Mitsuhiro Ebara: Conceptualization, resources, original draft writing, supervision, review & editing.

## Conflict of interest statement

The authors declare that they have no conflict of interest.

## Figures and Tables

**Table 1:**
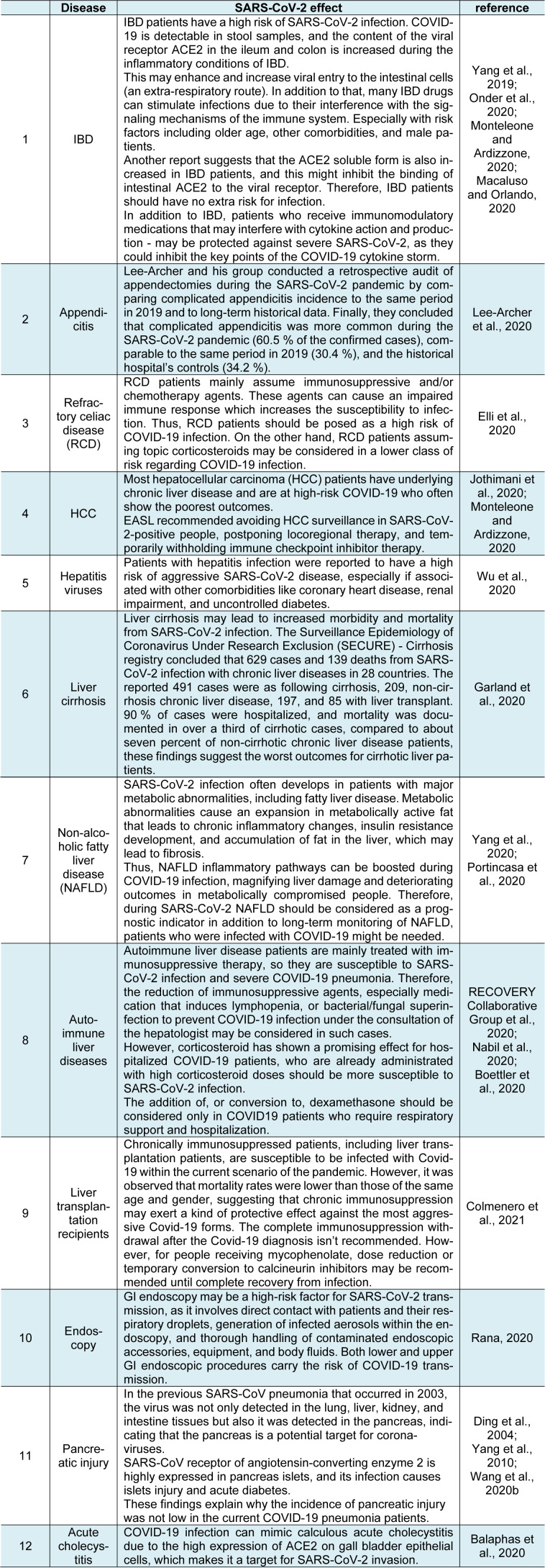
SARS-CoV-2 in gastrointestinal, liver, and pancreatic diseases

## References

[1] Aguila EJT, Cua IHY, Dumagpi JEL, Francisco CPD, Raymundo NTV, Sy-Janairo MLL (2020). COVID-19 and its effects on the digestive system and endoscopy practice. JGH Open.

[2] Balaphas A, Gkoufa K, Meyer J, Peloso A, Bornand A, McKee TA (2020). COVID-19 can mimic acute cholecystitis and is associated with the presence of viral RNA in the gallbladder wall. J Hepatol.

[3] Boettler T, Marjot T, Newsome PN, Mondelli MU, Maticic M, Cordero E (2020). Impact of COVID-19 on the care of patients with liver disease: EASL-ESCMID position paper after 6 months of the pandemic. JHEP Rep.

[4] Budden KF, Gellatly SL, Wood DL, Cooper MA, Morrison M, Hugenholtz P (2017). Emerging pathogenic links between microbiota and the gut-lung axis. Nat Rev Microbiol.

[5] Chen H, Ai L, Lu H, Li H (2020). Clinical and imaging features of COVID-19. Radiol Infect Dis.

[6] Cheung KS, Hung IFN, Chan PPY, Lung KC, Tso E, Liu R (2020). Gastrointestinal manifestations of SARS-CoV-2 infection and virus load in fecal samples from a Hong Kong Cohort: Systematic review and meta-analysis. Gastroenterology.

[7] Colmenero J, Rodríguez-Perálvarez M, Salcedo M, Arias-Milla A, Muñoz-Serrano A, Graus J (2021). Epidemiological pattern, incidence and outcomes of COVID-19 in liver transplant patients. J Hepatol.

[8] Ding Y, He L, Zhang Q, Huang Z, Che X, Hou J (2004). Organ distribution of severe acute respiratory syndrome (SARS) associated coronavirus (SARS-CoV) in SARS patients: implications for pathogenesis and virus transmission pathways. J Pathol.

[9] ECDC, European Centre for Disease Prevention and Control (2021). COVID-19 situation update worldwide, as of week 1 2021. https://www.ecdc.europa.eu/en/geographical-distribution-2019-ncov-cases.

[10] Elli L, Scaramella L, Lombardo V, Scricciolo A, Doneda L, Roncoroni L (2020). Refractory celiac disease and COVID-19 outbreak: Findings from a high incidence scenario in Northern Italy. Clin Res Hepatol Gastroenterol.

[11] EndCoronavirus (2021). Which countries do best in beating COVID-19?. https://www.endcoronavirus.org/countries.

[12] Garland V, Kumar AB, Borum ML (2020). Gastrointestinal and hepatic manifestations of COVID-19: Evolving recognition and need for increased understanding in vulnerable populations. J Natl Med Assoc.

[13] Giacomelli A, Pezzati L, Conti F, Bernacchia D, Siano M, Oreni L (2020). Self-reported olfactory and taste disorders in patients with severe acute respiratory coronavirus 2 infection: A cross-sectional study. Clin Infect Dis.

[14] Goldberg-Stein S, Fink A, Paroder V, Kobi M, Yee J, Chernyak V (2020). Abdominopelvic CT findings in patients with novel coronavirus disease 2019 (COVID-19). Abdom Radiol (NY).

[15] Grasselli G, Zangrillo A, Zanella A, Antonelli M, Cabrini L, Castelli A (2020). Baseline characteristics and outcomes of 1591 patients infected with SARS-CoV-2 addmitted to ICUs of the Lombardy Region, Italy. JAMA.

[16] Hamming I, Timens W, Bulthuis ML, Lely AT, Navis G, van Goor H (2004). Tissue distribution of ACE2 protein, the functional receptor for SARS coronavirus. A first step in understanding SARS pathogenesis. J Pathol.

[17] He L-H, Ren L-F, Li J-F, Wu Y-N, Li X, Zhang L (2020). Intestinal flora as a potential strategy to fight SARS-CoV-2 infection. Front Microbiol.

[18] Holshue ML, DeBolt C, Lindquist S, Lofy KH, Wiesman J, Bruce H (2020). First case of 2019 novel coronavirus in the United States. N Engl J Med.

[19] Hufnagl K, Pali-Schöll I, Roth-Walter F, Jensen-Jarolim E (2020). Dysbiosis of the gut and lung microbiome has a role in asthma. Semin Immunopathol.

[20] Jothimani D, Venugopal R, Abedin MF, Kaliamoorthy I, Rela M (2020). COVID-19 and the liver. J Hepatol.

[21] La Rosa G, Bonadonna L, Lucentini L, Kenmoe S, Suffredini E (2020). Coronavirus in water environments: Occurrence, persistence and concentration methods - A scoping review. Water Res.

[22] Lee-Archer P, Blackall S, Campbell H, Boyd D, Patel B, McBride C (2020). Increased incidence of complicated appendicitis during the COVID-19 pandemic. J Paediatr Child Health.

[23] Lin L, Jiang X, Zhang Z, Huang S, Zhang Z, Fang Z (2020). Gastrointestinal symptoms of 95 cases with SARS-CoV-2 infection. Gut.

[24] Macaluso FS, Orlando A (2020). COVID-19 in patients with inflammatory bowel disease: A systematic review of clinical data. Dig Liver Dis.

[25] Mao R, Qiu Y, He J-S, Tan J-Y, Li X-H, Liang J (2020). Manifestations and prognosis of gastrointestinal and liver involvement in patients with COVID-19: a systematic review and meta-analysis. Lancet Gastroenterol Hepatol.

[26] Monteleone G, Ardizzone S (2020). Are patients with inflammatory bowel disease at increased risk for Covid-19 infection?. J Crohn's Colitis.

[27] Nabil A, Uto K, Elshemy MM, Soliman R, Hassan AA, Ebara M (2020). Current coronavirus (SARS-CoV-2) epidemiological, diagnostic and therapeutic approaches: An updated review until June 2020. EXCLI J.

[28] Onder G, Rezza G, Brusaferro S (2020). Case-fatality rate and characteristics of patients dying in relation to COVID-19 in Italy. JAMA.

[29] Pan L, Mu M, Yang P, Sun Y, Wang R, Yan J (2020). Clinical characteristics of COVID-19 patients with digestive symptoms in Hubei, China: A descriptive, cross-sectional, multicenter study. Am J Gastroenterol.

[30] Portincasa P, Krawczyk M, Smyk W, Lammert F, Di Ciaula A (2020). COVID-19 and non-alcoholic fatty liver disease: Two intersecting pandemics. Eur J Clin Invest.

[31] Qi F, Qian S, Zhang S, Zhang Z (2020). Single cell RNA sequencing of 13 human tissues identify cell types and receptors of human coronaviruses. Biochem Biophys Res Commun.

[32] Rana SS (2020). Risk of COVID-19 transmission during gastrointestinal endoscopy. J Dig Endosc.

[33] RECOVERY Collaborative Group, Horby P, Lim WS, Emberson JR, Mafham M, Bell JL, (2020). Dexamethasone in hospitalized patients with Covid-19 - preliminary report. N Engl J Med.

[34] van Doremalen N, Bushmaker T, Morris DH, Holbrook MG, Gamble A, Williamson BN (2020). Aerosol and surface stability of SARS-CoV-2 as compared with SARS-CoV-1. N Engl J Med.

[35] Wang D, Hu B, Hu C, Zhu F, Liu X, Zhang J (2020). Clinical characteristics of 138 hospitalized patients with 2019 novel coronavirus-infected pneumonia in Wuhan, China. JAMA.

[36] Wang F, Wang H, Fan J, Zhang Y, Wang H, Zhao Q (2020). Pancreatic injury patterns in patients with coronavirus disease 19 pneumonia. Gastroenterology.

[37] WHO, World Health Organization (2020). Coronavirus disease (COVID-19) pandemic. https://www.who.int/emergencies/diseases/novel-coronavirus-2019?gclid=CjwKCAiAo5qABhBdEiwAOtGmbl0STs3tQBwkmtbstD2swrrAU3eRO8p1CRbdvXkNhq6McLVIkgpizBoCTEkQAvD_BwE.

[38] Wu J, Song S, Cao HC, Li LJ (2020). Liver diseases in COVID-19: Etiology, treatment and prognosis. World J Gastroenterol.

[39] Xiao F, Tang M, Zheng X, Liu Y, Li X, Shan H (2020). Evidence for gastrointestinal infection of SARS-CoV-2. Gastroenterology.

[40] Yang J-K, Lin S-S, Ji X-J, Guo L-M (2010). Binding of SARS coronavirus to its receptor damages islets and causes acute diabetes. Acta Diabetol.

[41] Yang S, Li X, Yang F, Zhao R, Pan X, Liang J (2019). Gut microbiota-dependent marker TMAO in promoting cardiovascular disease: Inflammation mechanism, clinical prognostic, and potential as a therapeutic target. Front Pharmacol.

[42] Yang X, Yu Y, Xu J, Shu H, Xia J, Liu H (2020). Clinical course and outcomes of critically ill patients with SARS-CoV-2 pneumonia in Wuhan, China: A single-centered, retrospective, observational study. Lancet Respir Med.

[43] Zhang H, Kang Z, Gong H, Xu D, Wang J, Li Z (2020). Digestive system is a potential route of COVID-19: An analysis of single-cell coexpression pattern of key proteins in viral entry process. Gut.

